# Efficient diagnosis of IDH-mutant gliomas: 1p/19qNET assesses 1p/19q codeletion status using weakly-supervised learning

**DOI:** 10.1038/s41698-023-00450-4

**Published:** 2023-09-16

**Authors:** Gi Jeong Kim, Tonghyun Lee, Sangjeong Ahn, Youngjung Uh, Se Hoon Kim

**Affiliations:** 1 Department of Pathology, Severance Hospital, Yonsei University College of Medicine, Seoul, Republic of Korea; 2 Department of Medicine, Yonsei University Graduate School, Seoul, Republic of Korea; 3 Department of Artificial Intelligence, Yonsei University College of Computing, Seoul, Republic of Korea; 4 Department of Pathology, Korea University Anam Hospital, Korea University College of Medicine, Seoul, Republic of Korea

**Keywords:** CNS cancer, Pathology, Mathematics and computing

## Abstract

Accurate identification of molecular alterations in gliomas is crucial for their diagnosis and treatment. Although, fluorescence in situ hybridization (FISH) allows for the observation of diverse and heterogeneous alterations, it is inherently time-consuming and challenging due to the limitations of the molecular method. Here, we report the development of 1p/19qNET, an advanced deep-learning network designed to predict fold change values of 1p and 19q chromosomes and classify isocitrate dehydrogenase (IDH)-mutant gliomas from whole-slide images. We trained 1p/19qNET on next-generation sequencing data from a discovery set (DS) of 288 patients and utilized a weakly-supervised approach with slide-level labels to reduce bias and workload. We then performed validation on an independent validation set (IVS) comprising 385 samples from The Cancer Genome Atlas, a comprehensive cancer genomics resource. 1p/19qNET outperformed traditional FISH, achieving *R*^2^ values of 0.589 and 0.547 for the 1p and 19q arms, respectively. As an IDH-mutant glioma classifier, 1p/19qNET attained AUCs of 0.930 and 0.837 in the DS and IVS, respectively. The weakly-supervised nature of 1p/19qNET provides explainable heatmaps for the results. This study demonstrates the successful use of deep learning for precise determination of 1p/19q codeletion status and classification of IDH-mutant gliomas as astrocytoma or oligodendroglioma. 1p/19qNET offers comparable results to FISH and provides informative spatial information. This approach has broader applications in tumor classification.

## Introduction

Glioma is the most common type of malignant neoplasm in the central nervous system (CNS), accounting for almost 80% of all CNS malignant tumors^1,2^. With the recent advances in molecular biological research, a paradigm shift in the diagnosis of CNS neoplasms has indeed occurred. The 2016 World Health Organization (WHO) Classification of Tumors of the CNS emphasized the importance of an integrated assessment that incorporates both histological features and genetic alterations in the diagnostic workup of patients with glioma^3^. Furthermore, the new 2021 WHO classification divides adult-type diffuse gliomas into three different groups based on the mutations and copy number alterations they harbor: (1) astrocytoma, isocitrate dehydrogenase (IDH)-mutant, (2) oligodendroglioma, IDH-mutant and 1p/19q codeleted, and (3) glioblastoma, IDH-wildtype^4^.

Having a strong correlation with oligodendroglial histology^5,6^, 1p/19q codeletion is critical to the differentiation of IDH-mutant gliomas. In addition, 1p/19q codeletion is consistently demonstrated to be a favorable prognostic factor in IDH-mutant gliomas due to its predictive value for higher treatment responses to adjuvant chemotherapy^7–9^. Although medical oncologists possess an interest in the 1p/19q status and molecular techniques for its detection are used widespreadly, identifying 1p/19q codeletion in glioma can be challenging in clinical practice^10^.

Fluorescence in situ hybridization (FISH) is used to assess chromosomal abnormalities present in various tumors, and is commonly considered to be the gold standard in the detection of 1p/19q codeletion^11–13^. Despite continuing popularity in the clinical field, FISH-based assessment of the 1p/19q status requires arduous interpretation, and thus there is considerable variability in its performance^12,14^. FISH also mandates the installation of special equipment, reagents, and a separate dark room for fluorescence experiments and microscopic observation^15^.

Recent progress in slide digitization and mathematical image processing has overcome the limitations of traditional molecular methods and improved the morphological analysis of pathological tissues^16–20^. This has greatly enhanced the clinical and research capabilities of pathology. Deep learning (DL) models have been proposed to extract meaningful image features within whole-slide images (WSIs), enabling clinicians to gain clinical and biological insights^21–23^. However, a common challenge arises when working with WSIs, as they tend to be exceptionally large and cannot be directly processed by neural networks. Typically, WSIs are divided into smaller patches, which are then fed into neural networks^24–27^. Nevertheless, this approach poses challenges: the patches within a WSI may have differing ground truth labels, and the sheer number of patches makes manual annotation difficult.

In our study, we present a novel and effective DL framework called 1p/19qNET. This framework is designed for predicting the 1p/19q status and diagnosing IDH-mutant gliomas within WSIs. To overcome the aforementioned limitations, we have adopted a weakly supervised learning approach, which leverages the fold change (FC) values of WSIs to guide the training process. Importantly, our method not only produces predictions but also offers insights by visually representing the estimated FC values on a patch-by-patch basis, providing explanations for the overall slide-wise FC values.

## Results

### Characteristics of the Study Cohort

In our discovery set (DS), a total of 288 patients were utilized to obtain complete digitalized histologic images and patient data. Supplementary Table 1 provides an overview of the clinicopathological characteristics of the study cohort. Notably, the comparison between astrocytoma and oligodendroglioma revealed statistically significant differences in age at surgery and CDKN2A/B status, as anticipated.

### FC prediction on the DS

**Fig. 1:**
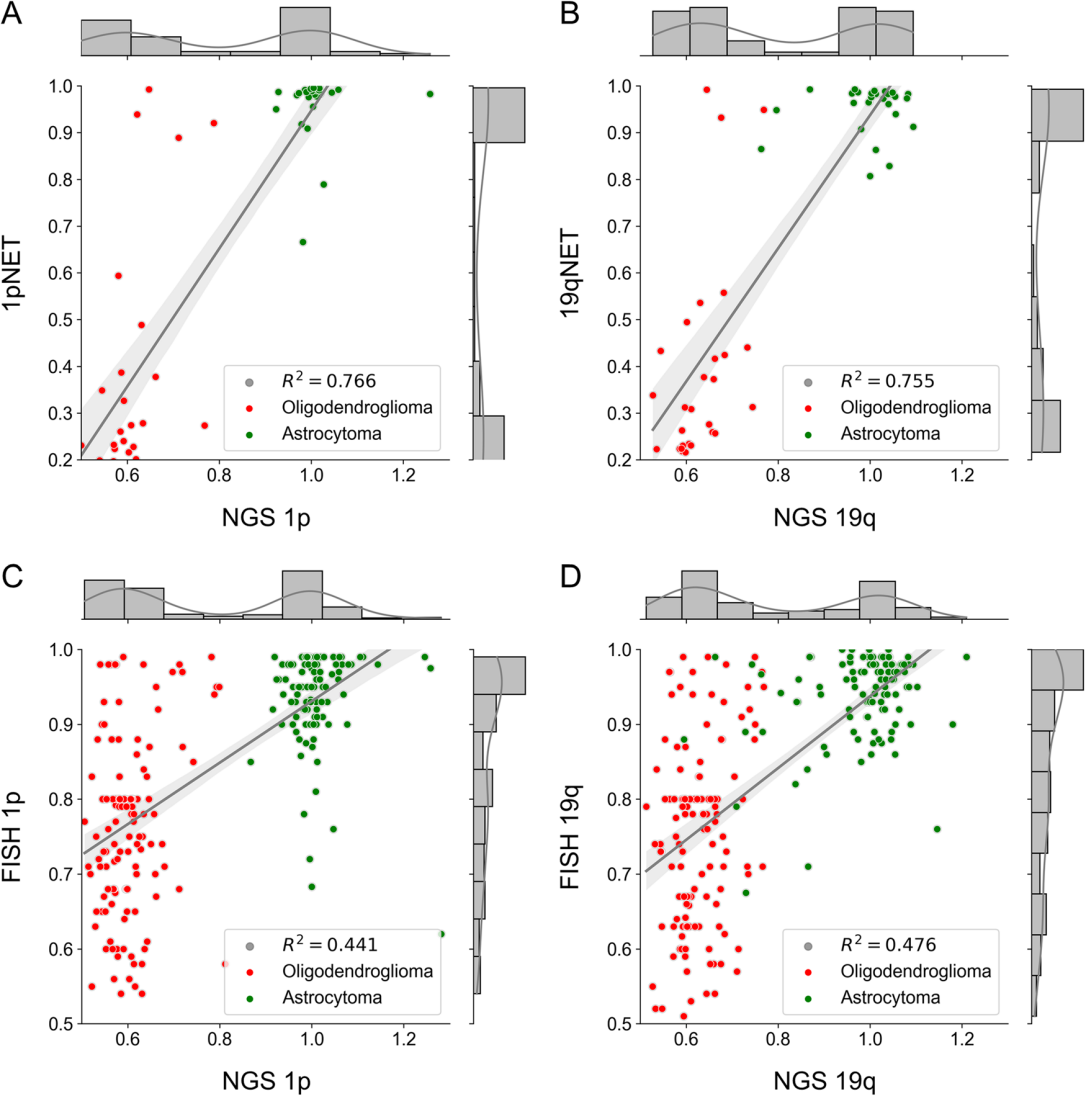
Comparison of NGS results with predicted fold change values from 1p/19qNET and signal ratio from FISH. **A**, **B** The results for 1pNET and 19qNET generated using the dataset with the best performance, respectively. Complete results are presented in Supplementary Table 2. **C**, **D** conventional FISH for 1p and 19q, respectively. Approaching a value of 1, *R*^2^ indicates a highly effective model. The overall average *R*^2^ was 0.589 for 1pNET and 0.547 for 19qNET. These values demonstrate that 1p/19qNET has a higher predictive power compared to FISH. The *R*^2^ values for 1p and 19q in FISH were 0.441 and 0.476, respectively. *R*^2^, coefficient of determination; NGS next-generation sequencing, FISH fluorescence in situ hybridization.

To ensure a reliable evaluation of the models’ performance, we employed a rigorous cross-validation approach on the DS. The dataset was randomly split into three sets: training (60%), validation (20%), and test (20%). We conducted 10-fold cross-validation and reported the average performance metrics. The term “FC value” represents the expression level of genes located on the 1p or 19q chromosome, which is standardized to 1 in normal tissue. It quantifies the degree of increase or decrease in expression compared to the normal tissue. For astrocytoma, which does not exhibit 1p/19q codeletion, the FC value would be close to 1. However, in oligodendroglioma, where the presence of 1p/19q codeletion is a diagnostic criterion, the FC value would be decreased, ranging from 0.5 to 0.8, depending on tumor purity. The 1p/19qNET system enabled the prediction of FC values as a continuous variable, allowing for slide-level predictions on the DS to be evaluated through linear regression. The plots shown in Fig. 1 compare the next-generation sequencing (NGS) results with the predicted FC values obtained from a representative fold of 1p/19qNET and the signal ratio obtained from FISH. Upon observation, it can be seen that the dispersion of the test sets obtained by 1p/19qNET has a better-centered regression line compared to that of FISH. The *R*^2^ values of slide-level predictions of 1p/19qNET across the 10 folds were found to be 0.589 for the 1p arm and 0.547 for the 19q arm, respectively. In comparison, the *R*^2^ values obtained from FISH were lower, with 0.441 for the 1p arm and 0.476 for the 19q arm, respectively. This indicates that the FC predictions made by 1p/19qNET are generally more consistent across WSIs, and exhibit superior predictive power for 1p/19q status compared to the traditional FISH method. Additionally, the FC prediction values generated by 1p/19qNET exhibit notable discrepancies in their distribution between oligodendroglioma and astrocytoma. Specifically, the average FC prediction value on 1pNET for oligodendroglioma is 0.502 ± 0.085 and 0.932 ± 0.027 for astrocytoma (p < .001 by two-tailed *t*-test) and that on 19qNET for oligodendroglioma is 0.524 ± 0.078 and 0.932 ± 0.029 for astrocytoma (*p* < 0.001 by two-tailed *t*-test). The results are summarized in Supplementary Table 2.

### Tumor type prediction on the DS and independent validation set

**Fig. 2:**
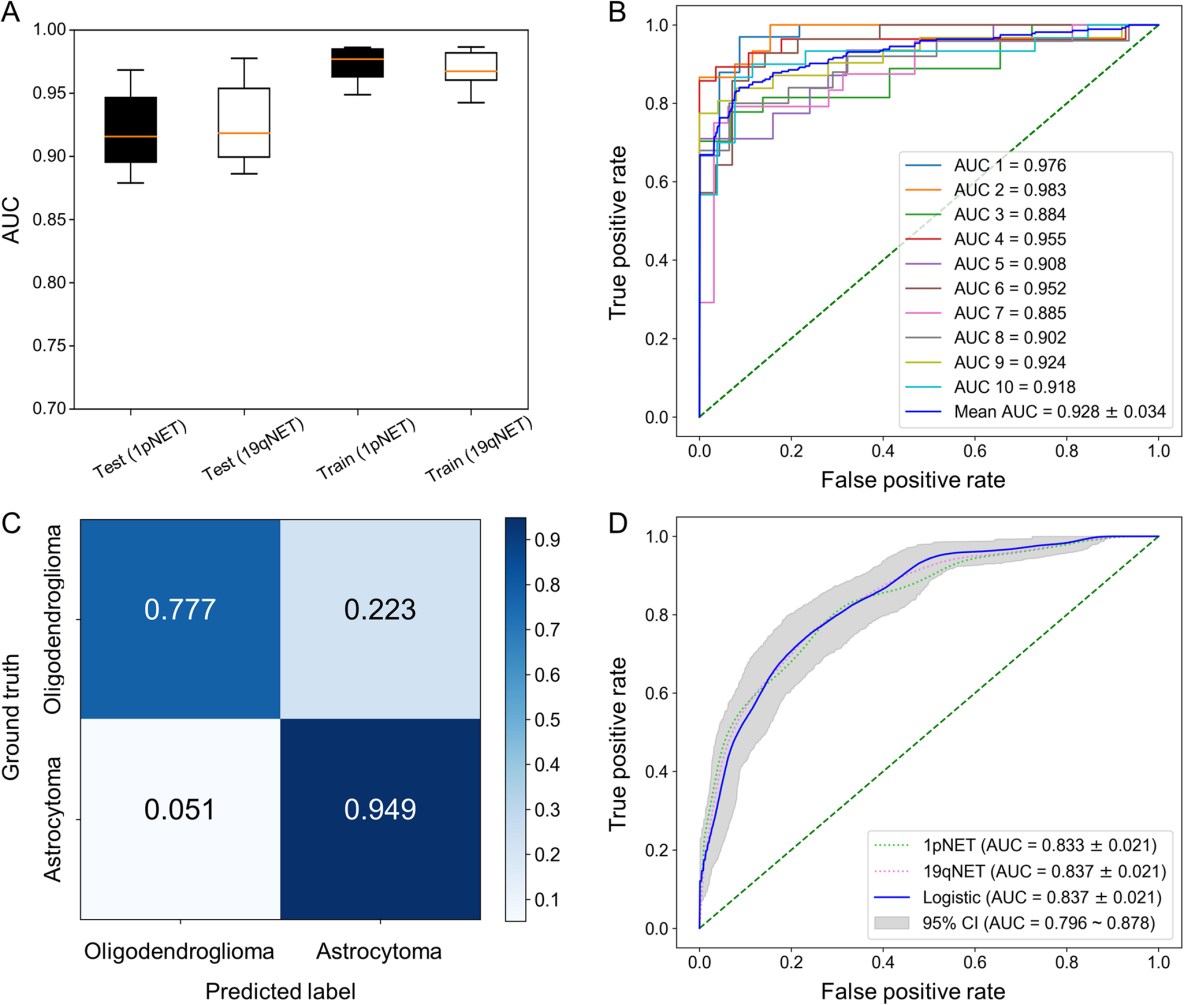
Diagnostic performance of 1p/19qNET. **A** boxplot of the AUC for 1pNET and 19qNET on the train and test sets. Both 1pNET and 19qNET maintained excellent diagnostic performance on the test set. Boxes indicate interquartile range, lines are medians, and whiskers extend to 1.5 the interquartile range. **B** ROC curves of 1p/19qNET on each fold of the discovery set. The logistic model combining 1pNET and 19qNET was validated using 10-fold cross-validation to assess its ability to accurately distinguish IDH-mutant gliomas. All 10 individual results consistently demonstrated a performance worthy of recognition. **C** confusion matrix of 1p/19qNET. **D** ROC curves of 1p/19qNET on the independent validation set with bootstrap-confirmed CI of the logistic model. AUC area under the curve, CI confidence interval, ROC receiver operating characteristic, IDH isocitrate dehydrogenase.

In the DS, both 1pNET and 19qNET exhibited remarkable discriminatory capacity for glioma, leveraging FC prediction as a basis. The average AUC values in the test sets, as depicted in Fig. 2A and Supplementary Table 2, further reinforced their diagnostic prowess, measuring 0.921 and 0.927, respectively. The logistic model was created to differentiate gliomas by combining the results of copy number loss in 1pNET and 19qNET, which was similar to the process of differentiating gliomas using FISH-based detection in actual clinical environments. This model demonstrated excellent performance from a statistical standpoint (AUC = 0.930, Fig. 2B and Supplementary Fig. 1). In fact, its performance was comparable to the accuracy (0.861 vs 0.843) and F1 score (0.850 vs 0.831) of the results obtained by FISH (Fig. 2C). The statistical findings are detailed in Table 1.

**Table 1:** Diagnostic predictive ability of 1p/19qNET on discovery set and independent validation set.

	Accuracy	Precision	Recall	F1-Score	AUC
Discovery set					
1pNET	0.884	0.929	0.840	0.879	0.921
19qNET	0.891	0.940	0.841	0.885	0.927
Logistic model	0.861	0.944	0.776	0.850	0.930
Conventional FISH	0.843	0.978	0.722	0.831	—
Independent validation set					
1pNET	0.777	0.725	0.706	0.715	0.833
19qNET	0.766	0.684	0.765	0.722	0.837
Logistic model	0.725	0.831	0.386	0.527	0.837

*AUC* area under the curve, *FISH* fluorescence in situ hybridization.

An additional statistical analysis was conducted to determine whether 1p/19qNET is influenced by neoadjuvant therapy or previous surgical history, or if it exhibits vulnerability to tumor grade. The classification accuracy for cases that previously underwent chemoradiation therapy was found to be 0.850, which was not significantly different from the overall accuracy of 0.861. When analyzing the data by classifying based on the tumor grade (Grade 2: 0.834, Grade 3: 0.875, Grade 4: 0.886), no significant differences were observed in the model’s performance.

Following pre-processing, 153 oligodendroglioma and 232 astrocytoma patients were available for analysis. We trained 1p/19qNET using all the slides included in the DS and evaluated its performance on the independent validation set (IVS) slides. Impressively, the logistic model achieved good discrimination between astrocytoma and oligodendroglioma, without the need for clinical information or laborious annotation by human experts. The logistic model obtained an AUC of 0.837 (95% confidence interval: 0.796–0.878) and 1pNET and 19qNET also demonstrated similar performance. Detailed statistical results are presented in Table 1, while the corresponding ROC curves are displayed in Fig. 2D and Supplementary Fig. 2.

### Interpretability of 1p/19qNET

**Fig. 3:**
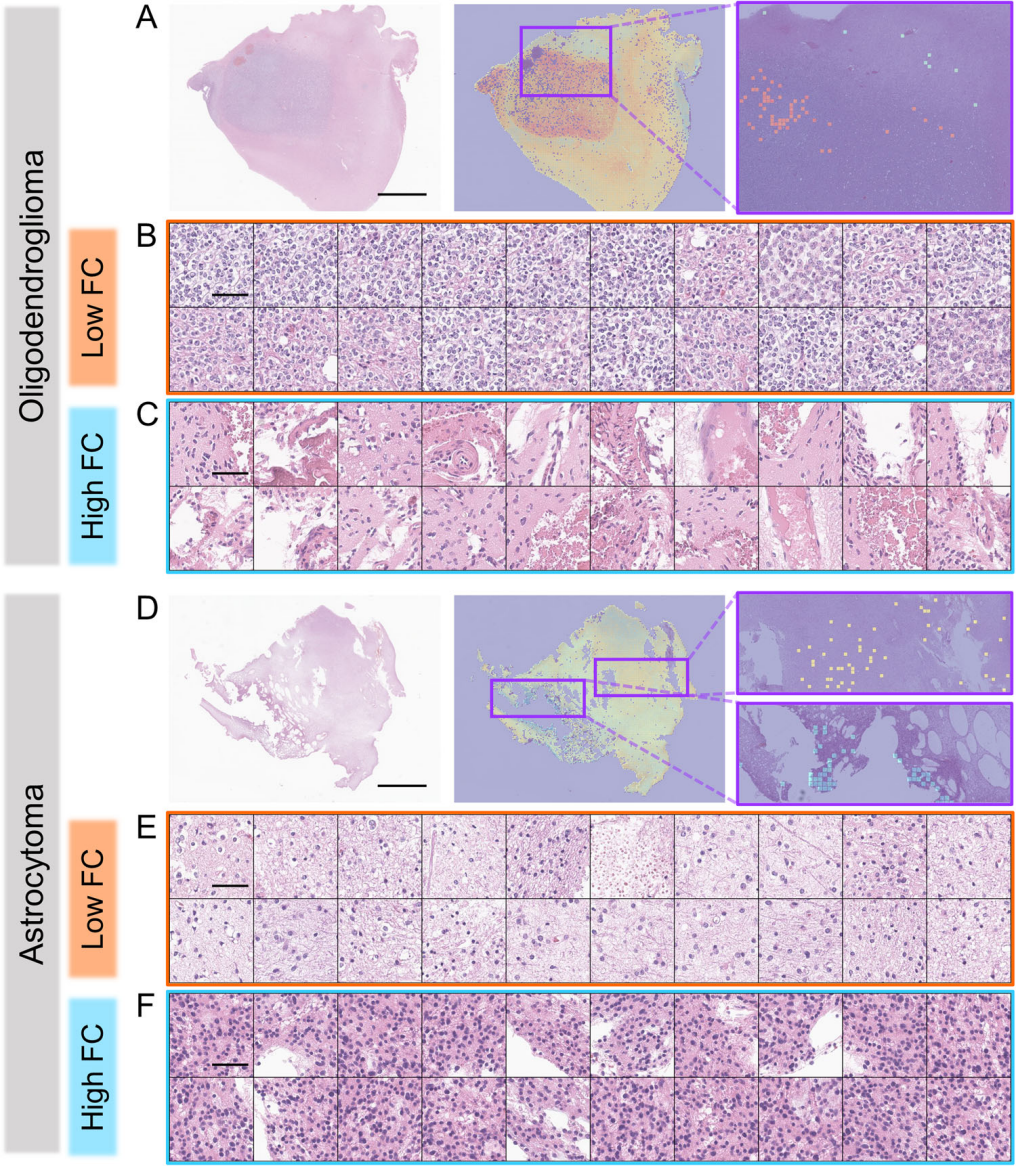
Heatmaps and representative patches of 1pNET. **A**–**C** Oligodendroglioma. **D**–**F** astrocytoma. **A**, **D** heatmap and distribution of representative patches. Scale bar, 5 mm. **B**, **E** 20 patches out of 100 representative patches with low FC value. Scale bar, 50 μm. **C**, **F** 20 patches out of 100 representative patches with high FC value. Scale bar, 50 μm. FC fold change.

**Fig. 4:**
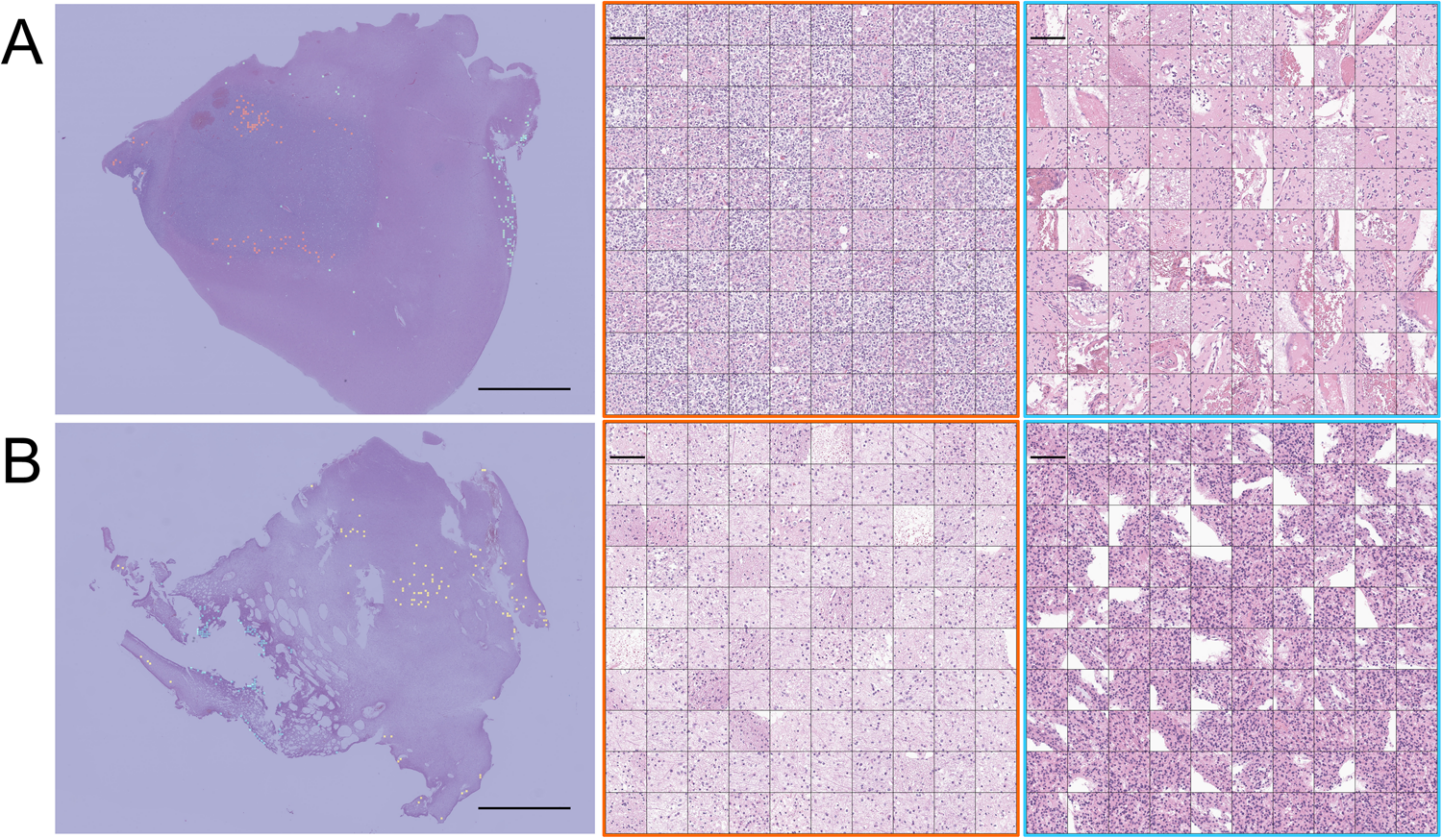
Patch anthology of 1pNET. For each case, (**A**) Oligodendroglioma and (**B**) astrocytoma, the collection consisted of 200 representative patches that were selected specifically for 1pNET. Left, distribution of representative patches. Scale bar, 5 mm.; middle, all representative patches with low FC value. Scale bar, 100 μm.; right, all representative patches with high FC value. Scale bar, 100 μm. FC fold change.

To investigate the interpretability of 1p/19qNET, we generated heatmaps for all patients’ WSIs. In Fig. 3, we have shown that there is significant variability in the FC prediction values within and across the WSIs, suggesting that the histologic features associated with FC, as learned by the 1p/19qNET, are heterogeneously distributed in the hematoxylin and eosin (H&E) slides. Upon closer examination, we confirmed the presence of features that help predict 1p/19q codeletion in IDH-mutant glioma. Specifically, in patches with low FC values in oligodendroglioma, we observed round nuclei with mild to moderate nuclear atypia, perinuclear clearing, and distinct cell borders. In contrast, those with high FC values exhibited reactive gliosis with low cellularity. Interestingly, in cases predicted to be astrocytoma, even patches with low FC values did not reveal histologic features suggestive of oligodendroglioma; instead, they showed tumoral or non-tumoral areas with some degree of hypercellularity. Conversely, patches with high FC values had variable cellular morphology, revealing oval to elongated nuclei and fibrillar glial processes, which could support a diagnosis of astrocytoma. Figure 4 and Supplementary Fig. 3 provide additional details.

## Discussion

This study aims to contribute to the ongoing advancements in DL-based diagnostics, in which molecular research on diseases and the performance of artificial intelligence-driven technologies has provided a strong impact^28–30^. The new 2021 WHO classification highlights the importance of IDH mutation and 1p/19q codeletion in the diagnosis of adult-type diffuse gliomas, which prompted us to focus on these two genetic abnormalities^3–5^. Our hypothesis was that 1p/19qNET could extract meaningful features from H&E slides to detect 1p/19q codeletion, without requiring laborious efforts to define the complicated, even impossible, boundaries of gliomas. To test our hypothesis, we began with establishing a DS comprising digitalized WSIs of glioma, corresponding FISH-based results of the 1p/19q status, and FC values of 1p and 19q arms confirmed by NGS. We then assessed whether 1p/19qNET could properly predict 1p/19q status using only slide-level labels, rather than patch-level annotations. 1p/19qNET, as anticipated, demonstrated high *R*^2^ values (0.589 and 0.547) in the linear regression analysis, which were superior to those (0.441 and 0.476) obtained using conventional FISH.

The obtained results go beyond linear regression, as they can reach a diagnostic performance that is as highly reliable as that of conventional FISH. It is widely acknowledged that distinguishing between astrocytoma and oligodendroglioma is challenging due to their mixed features, which makes the classification of low-grade glioma extremely difficult^31–33^. Despite this difficulty, 1pNET and 19qNET can discriminate oligodendroglioma from astrocytoma, exhibiting high AUCs of 0.921 and 0.927, respectively. These two networks can be combined to create a merged prediction, as if it were virtual FISH, and the logistic regression encompassing 1pNET and 19qNET achieved an AUC of 0.930 in the DS. Interestingly, this is precisely the process that clinicians undertake when carrying out FISH analyses. This even holds when examining the diagnostic ability of 1p/19qNET on the IVS, where it obtained an AUC of 0.837 in differentiating oligodendroglioma from astrocytoma. In contrast to earlier investigations on differentiating glioma using DL^32,34,35^, the 1p/19qNET method does not rely on clinical and pathological data as an input throughout the entire process. Additionally, the model’s robustness was demonstrated on the IVS. Moreover, each case underwent validation through NGS and corresponding copy number plots, instilling a sense of trust in the outcomes. As far as our knowledge extends, this is the first DL study on gliomas that validates genetic anomalies in all cases through NGS.

FISH is a molecular technique for detecting and locating specific deoxyribonucleic acid sequences on chromosomes^36^. It is considered the gold standard method for detecting 1p/19q codeletion in glioma and can indicate where the fluorescent probe is bound^11^. Being widely used in clinical practice, FISH has significant drawbacks that cannot be ignored. It necessitates a high labor input and additional resources, such as a separate dark room to conduct fluorescence experiments, special equipment, solution, and reagents^15^. Moreover, the time-consuming nature of interpretation and analysis, coupled with considerable variability in FISH results on 1p/19q status, further complicates its use in clinical practice^12,14,37^. It is possibly introduced by an unavoidable bias due to the random selection of tumor cells, which can reduce diagnostic accuracy, especially in heterogeneous cases with a high proportion of non-tumor cells^38^.

DL models, once trained, can provide equivalent or better performance than traditional diagnostic methods while reducing the time and effort required for diagnosis^39^. In fact, 1p/19qNET, for example, can predict the FC values of the 1p and 19q arms and provide a suggestive diagnosis within just a few minutes per WSI. Furthermore, this model is an attractive option in the field of digital pathology, where even experts may face difficulty annotating slides, as it requires no special equipment or human intervention after scanning H&E slides. Although fully supervised methods are still widely used in DL-based digital pathology^40,41^, the sparsity of patch-level annotations and the significant time required to generate them can limit their practicality. Additionally, the ambiguity of tissue boundaries can lead to discrepancies among experts, ultimately undermining the robustness of the model. To protect a model from these drawbacks and ensure stable learning, one of the most reliable approaches currently available is to use a weakly-supervised learning approach to enable the model to directly identify meaningful features, as is the case in this study. 1p/19qNET model predicts tumor-related information and automatically generates visual evidence to support its decisions without relying on expert annotations, which helps to minimize the impact of personal biases on the model’s predictions.

Visualization of DL methods is imperative for experts to perceive their results and helps clinical practice, especially in the field of pathology^42^. We succeeded in visualizing FC predictions of 1p/19qNET across the entire patches in all WSIs and presented an innovative approach that enables subsequent analysis by experienced pathologists. Techniques analogous to our visualization used in this work can further develop researchers’ biological understanding of tumors by not only presenting previously known histologic features, but also discovering new ones related to molecular changes that only DL can identify. It is worth noting, however, that while DL models that provide reliable visualizations show great promise for future development in clinical situations where molecular testing is often limited, continuous research and verification in diverse settings are necessary to further improve their accuracy and effectiveness. Therefore, ongoing efforts to refine and validate these models are crucial to ensure their reliability and usefulness in real-world clinical scenarios.

Despite our encouraging achievements, several factors have limited the contributions of our work. First, we were unable to analyze FC values on the IVS. Although the performance that 1p/19qNET exhibited was as high as expected, it remains unclear whether its achievement was obtained by the way the authors intended. Second, we did not exhaustively explore other approaches that could potentially improve the accuracy of prediction at the patient level by combining the results of 1pNET and 19qNET. This implies that there is still considerable room for improvement in this area beyond the logistic regression method. By addressing this, we anticipate that 1p/19qNET can surpass the diagnostic outcomes achieved even when incorporating p53 and ATRX immunostaining findings. Finally, this study was based on a relatively small dataset from a single tertiary institution and had a retrospective study design. Therefore, the findings of this study should be verified and extended in future prospective clinical studies.

In summary, our study details the successful application of DL-based estimation in accurately determining 1p/19q codeletion and diagnosing IDH-mutant gliomas as either astrocytoma or oligodendroglioma. Notably, our 1p/19qNET approach, which relies solely on slide-level labels, delivers comparative performance to conventional FISH-based methods and autonomously presents informative locations. Encouragingly, our model exhibits diagnostic robustness on an IVS, bolstering the flexibility and reliability of this framework for clinical decision-making and cancer research. DL-based estimation holds significant potential to streamline diagnosis and tailor patient therapy, reducing both time and effort for clinicians.

## Methods

### Study population and digitization protocol

The DS in this study was obtained from surgical resections of diffuse glioma patients who received treatment at Severance Hospital between May 2017 and December 2022. Detailed clinical data, such as age, sex, patient history, and tumor grade, were retrieved from the patients’ medical records. This study was approved by the Institutional Review Board of Severance Hospital, Seoul, Korea, with the waiver for written informed consent (IRB no. 4-2022-1493). The IVS used in this study consisted of patients from The Cancer Genome Atlas Merged Cohort of LGG and GBM (TCGA-LGGGBM), which is a publicly available multi-institutional dataset. For both cohorts, patients presenting with IDH-wildtype glioma or with low-purity samples were omitted from the analysis. Additionally, cases in the TCGA-LGGGBM dataset that did not have a diagnostic permanent slide, but only frozen slides, were also excluded from the analysis.

The study coordinator carefully selected one representative H&E slide from each glioma case in the DS with the aim of utilizing it for both NGS and digitization purposes. Subsequently, the DS was digitized using a whole-slide scanner (Aperio GT 450, Leica Biosystems Imaging, Inc., Vista, CA, USA). During the independent validation phase, the pathologists G. J. K. and S. H. K. handpicked a WSI image from each patient in the TCGA-LGGGBM dataset. Both the images from the DS and the IVS were scanned with a 40x objective, yielding a tissue length of 0.26μm per pixel side and 0.25μm per pixel side, respectively.

### Data pre-processing

WSIs were divided into non-overlapping patches of 224 × 224 pixels at a magnification of 20x objective. Among them, background patches and blood-containing patches were discarded since they were irrelevant to the diagnosis. The background patches were identified by counting the number of edge pixels, as the textureless backgrounds contained edge pixels less than 23. Blood-containing patches were identified by using the color threshold. Specifically, we converted each patch image from the RGB color space to the HSV color space, and then set the lower and upper bounds of the hue and saturation channels to detect patches with hues and saturations of blood stains.

To reduce the overhead of loading a large number of patches to GPU memory, we extracted the feature vectors in advance and stored the features instead of feeding the patches to the feature extractor on-the-fly. This approach was particularly useful, since the aggregator receives FC values of multiple patches in a WSI such that extracting features of multiple patches is memory-intensive. Furthermore, we applied ImageNet normalization to our DS when extracting features from the patches. However, due to uncontrollable factors, such as staining processes and scanners, the IVS exhibited color variations across slides scanned in different hospitals. To compensate for these variations and ensure consistency across the dataset, we also applied an additional color normalization technique to the IVS to match its color distribution to that of DS^43^.

### 1p/19qNET framework

**Fig. 5:**
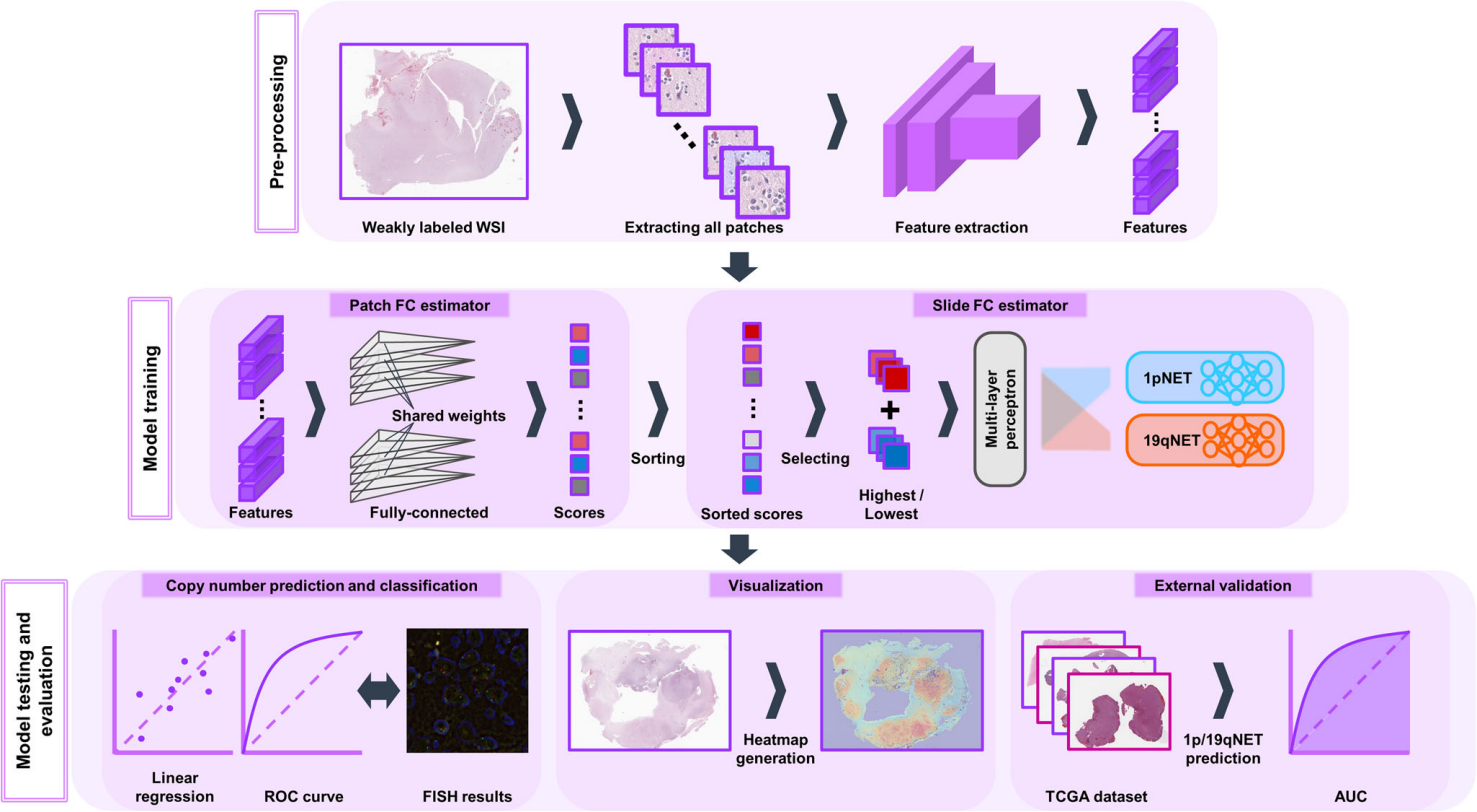
Patch anthology of 1pNET. With our proposed neural network model, we aimed to estimate the fold change values of glioma in a weakly-supervised manner by feeding the model with both a WSI and its corresponding slide-level label. We compared the model’s prediction power to that of conventional FISH and visualized its prediction clues for individual cases to enhance interpretability. To validate the robustness of our neural network model, we performed an independent validation on a public dataset. WSI whole-slide image, FC fold change, ROC receiver operating characteristic, FISH fluorescence in situ hybridization, TCGA The Cancer Genome Atlas, AUC area under the curve.

Our framework aimed to predict the 1p/19q status and diagnose glioma on WSIs through a neural network. The framework consisted of three main steps: feature extraction, FC value estimation, and tumor type diagnosis. In the feature extraction step, WSIs were encoded as feature vectors. Next, the FC estimator estimated the FC values of 1p and 19q from the extracted features. Finally, the 1p/19qNET model diagnosed the tumor types based on the estimated FC values. Figure 5 provides an overview of our framework.

As WSIs are much larger than conventional inputs to neural networks, we divided each WSI into non-overlapping patches and fed individual patches to the feature extractor. We adopted the RetCCL^44^, which has been pretrained on a diverse range of histopathological images, including TCGA and PAIP datasets. RetCCL effectively processed each patch and produced a feature vector of 2048 dimensions in its penultimate layer. Its network parameters were kept frozen.

Due to tumor heterogeneity, different histopathological features indicative of 1p/19q codeletion can vary across different regions of the tumor, even within a WSI. Hence, our FC estimator first produced patch-level FC values for each individual patch and predicted slide-level FC values from them. Similar to the CHOWDER model^24,45,46^, we arranged the highest N and lowest N values to feed them to a multi-layer perceptron (MLP) that produces the slide-level FC value. Then, the individual patches got the proper supervision on the patch-level FC values to correctly predict the slide-level FC value. The intuition behind this was similar to the way pathologists determine the tumor types by considering both supportive and contradictory histopathological features for the diagnosis. It also helped reduce the GPU memory footage by limiting the number of input patches. We employed a linear layer to estimate the patch-level FC values from feature vectors and a three-layer MLP with sigmoid activation after each layer to predict the slide-level FC values by aggregating the patch-level FC values. The three layers in the MLP had 200, 100, and 1 channels, respectively. Our framework had two FC estimators to predict the 1p and 19q FC values.

Finally, we combined the 1p and 19q FC values using a logistic regression model from scikit-learn’s linear model. This logistic regression model performed a linear combination of slide-level FC values, followed by a sigmoid activation function. The output of this model represented the probability of the WSI being diagnosed as either astrocytoma or oligodendroglioma.

To train our model, we used the mean squared error between the WSI-level FC estimates and the two ground truth scores acquired from NGS for chromosomes 1 and 19 as the loss function for the FC estimator. The final linear layer was trained to minimize the binary cross-entropy loss to predict tumor types.

### Training protocol and implementation details

Our model shares key hyperparameters with CHOWDER^24^, including the optimizer, learning rate, weight decay, and dropout probability. However, we made distinct choices for these hyperparameters. Specifically, we opted for the Adam optimizer with a learning rate of 0.0001 and a weight decay of 0.0005 during the training process. Furthermore, we set the dropout probability to 0.5 for the linear layers. It took 1.2 h to train each model using an NVIDIA RTX A5000. The optimal value for the hyperparameter N, which represents the number of highest or lowest-value patches fed into the MLP for slide-level FC prediction, was found to be 100, resulting in the best performance.

### Explaining the diagnosis by visualization

Our model assigns a FC value to each patch on the WSI, reflecting its 1p/19q status. Patches indicating 1p/19q loss are colored in red, those indicating intact 1p/19q are colored in blue, and the omitted patches containing background or blood are colored in purple. The results of each patch are combined to provide a heatmap for a WSI, and the location information of representative patches with 100 highest or lowest values are also provided.

### FISH

Out of 288 patients, 236 who received resection between May 2017 and December 2021 underwent FISH-based detection to identify 1p/19q codeletion. Dual-color locus-specific identifier (LSI) probes targeting 1p36/1q25 and 19q13/19p13 (Vysis/Abbott Molecular Inc., IL, USA) were used to assess 1p/19q codeletion. The LSI 1p36 probe encompasses sequences starting near the SHGC‑57243 locus, passing through the TP73 and MEGF6 genes, and concluding just beyond the MEGF6 locus. Meanwhile, the LSI 1q25 probe includes sequences beginning past the ABL2 gene, traversing the ABL2 and ANGPTL1 genes, and terminating near the SHGC-1322 locus. Shifting to the LSI 19p13 probe, it involves sequences originating just centromeric to the MAN2B1 locus, proceeding through the MAN2B1, ZNF443, and ZNF44 genes, and coming to an end just past the ZNF44 locus. Finally, the LSI 19q13 probe comprises sequences that commence beyond the CRX locus, continue through the CRX, GLTSCR2, and GLTSCR1 genes, and conclude proximally to the GLTSCR1 locus. All probe pairs were co-denatured with the tissue sections and hybridized overnight at 37 °C in separate slides. After hybridization, the slides were washed on 2XSSC/0.1%NP-40 for 2 min at 73 °C, counterstained with 4′,6′-diamidino-2-phenylindole dihydrochloride, and then cover-slipped. The proportion of nuclei containing only one signal of 1p or 19q was calculated by evaluating more than 60 nuclei possessing two centromeric signals. Deletion was defined as a signal ratio of more than 50% for the region of interest compared to the control probe^47^.

### Mutational and copy number analysis using NGS

All cases included in the study underwent NGS to detect IDH1/2 mutation and confirm chromosome 1p/19q status. All cases included in the study underwent NGS to detect IDH1/2 mutation and confirm chromosome 1p/19q status. For NGS analysis, we used the Illumina TruSight Oncology 500 panel (Illumina, Milan, Italy) according to the manufacturer’s instructions. The gene panels covered 523 genes for both mutational analysis and copy number analysis, as listed in Supplementary Table 3. To perform mutational analysis, FASTQ files were uploaded on Illumina’s BaseSpace software for variant interpretation. Only variants in coding regions and promoter regions or splice variants were retained. In addition, only variants that were present in less than 1% of the population according to ExAC and 1000 Genomes databases or in more than 5% of reads with a minimum read depth of 250 were retained. IDH1/2 mutation status was reviewed, and only pathogenic variants were selected. For copy number analysis, we collected log2 FC values of the target genes across the chromosomes 1p and 19q arms, and calculated the mean values for each chromosome. The locations of the target genes are shown in Supplementary Figure 4. Based on our experience, we classified tumors with both mean values less than 0.8 as oligodendroglioma and those with at least one mean value greater than 0.8 as astrocytoma. To facilitate genome interpretation when the log2 FC values of the genes and their mean values were at the borderline, we referred to the copy number plots of the entire genome. Representative plots are shown in Supplementary Figure 5.

### Statistical methods

Clinicopathological characteristics were compared using appropriate statistical tests, including chi-square or Fisher’s exact test for categorical variables and t-test for continuous variables. The performance of the models was evaluated by various metrics. AUC values were calculated for 1pNET and 19qNET, as well as for the logistic regression model used to combine the results from both models. The best cut-off values for 1p/19qNET and FISH were used to construct confusion matrices; and accuracy, precision, recall, and F1 scores were calculated from these matrices. The extent to which the independent variables accounted for the variation in the dependent variable was measured using *R*^2^, which takes values between 0 and 1 (0 < *R*² < 1), with a value approaching 1 indicating a highly effective model in explaining the variation in the dependent variable based on independent variables.

To obtain more accurate estimates of the AUCs, we performed 1000 bootstraps for each test data split. This involved repeatedly resampling the data to create new datasets. The results of each bootstrap were then used to calculate the 95% confidence interval, which provided a reliable assessment of the models’ performance. We considered differences to be statistically significant if the two-sided *p*-value was <0.050. The data were analyzed using Python 3.11.2 from 2 January 2023 to 8 July 2023.

### Reporting summary

Further information on research design is available in the Nature Research Reporting Summary linked to this article.

### Supplementary information

SUPPLEMENTAL MATERIAL

Reporting Summary

## Data Availability

We conducted independent validation using a publicly available dataset accessible through the TCGA portal (https://portal.gdc.cancer.gov). Specifically, we utilized data from the TCGA-LGG and TCGA-GBM projects. The primary dataset that underpins the findings of this study is sourced from Severance Hospital. However, it’s important to note that access to this dataset is subject to restrictions, and its use in this study was permitted with specific authorization. As a result, this dataset is not accessible to the public. If required, access to the data or a subset for testing purposes may be granted by Severance Hospital pending the approval of ethical considerations.

## Code availability

The code used in this study is available at https://github.com/rogo96/1p19qNET. The source code and pretrained models for this study are available to the corresponding author upon academically reasonable request.
