# Supplementary material for: Efficient diagnosis of IDH-mutant gliomas: 1p/19qNET assesses 1p/19q codeletion status using weakly-supervised learning
Source: NPJ Precis Oncol. 2023 Sep 16;7:94. doi: 10.1038/s41698-023-00450-4 (PMC10505231; doi:10.1038/s41698-023-00450-4)
Supplement: Supplementary file 1 — SUPPLEMENTAL MATERIAL [file 41698_2023_450_MOESM1_ESM.pdf]

Supplementary Table 1. Clinicopathological Characteristics of Discovery Set

	**Astrocytoma (N=138)**	**Oligodendroglioma (N=150)**	**P-Value**
**Sex**			.369
Female	58 (42.0)	72 (48.0)	
Male	80 (58.0)	78 (52.0)	
**Age**	40.0 ± 11.0	44.4 ± 12.0	**<.001**
**Treatment history**			.524
No prior treatment	113 (81.9)	130 (86.7)	
Neoadjuvant therapy only	3 (2.2)	2 (1.3)	
Previous surgery with adjuvant therapy	22 (15.9)	18 (12.0)	
**Grade**			N/A
2	61 (44.2)	45 (30.0)	
3	38 (27.5)	105 (70.0)	
4	39 (28.3)	N/A	
**IDH mutation**			.055
IDH1	136 (98.6)	140 (93.3)	
IDH2	2 (1.4)	10 (6.7)	
**CDKN2A/B homozygous deletion**			**.002**
Absent	120 (87.0)	146 (97.3)	
Present	18 (13.0)	4 (2.7)	

[a] Values are presented as number (%) or mean ± standard deviation.
[b] Statistical analysis was conducted using Chi-square or Fisher's exact test for categorical variables and two-tailed t-test for continuous variables.
IDH, isocitrate dehydrogenase; CDKN, cyclin-dependent kinase inhibitor; N/A, not applicable.

Supplementary Table 2. Fold Change Prediction of 1p/19qNET and FISH on Discovery Set

	1pNET				19qNET				FISH 1p			FISH 19q		
	O	A	R^2	AUC	O	A	R^2	AUC	O	A	R^2	O	A	R^2
**Fold 1**	0.499	0.947	0.661	0.968	0.476	0.959	0.654	0.978						
**Fold 2**	0.464	0.949	0.655	0.949	0.566	0.958	0.563	0.955						
**Fold 3**	0.524	0.948	0.600	0.886	0.559	0.939	0.492	0.886						
**Fold 4**	0.365	0.961	0.766	0.955	0.395	0.952	0.755	0.949						
**Fold 5**	0.641	0.904	0.522	0.915	0.619	0.871	0.421	0.899						
**Fold 6**	0.447	0.919	0.522	0.940	0.467	0.940	0.602	0.957						
**Fold 7**	0.617	0.924	0.499	0.879	0.596	0.900	0.422	0.892						
**Fold 8**	0.484	0.935	0.618	0.903	0.487	0.955	0.588	0.899						
**Fold 9**	0.403	0.870	0.533	0.893	0.436	0.901	0.539	0.928						
**Fold 10**	0.580	0.961	0.518	0.917	0.634	0.946	0.436	0.909						
**Total**	**0.502 ±0.085**[a]	**0.932 ±0.027**[a]	**0.589**	**0.921**	**0.524 ±0.078**[a]	**0.932 ±0.029**[a]	**0.547**	**0.927**	**0.765 ±0.130**[a]	**0.937 ±0.130**[a]	**0.441**	**0.745 ±0.136**[a]	**0.939 ±0.137**[a]	**0.476**

[a] Values are presented as mean ± standard deviation. Statistical analysis was conducted using the two-tailed t-test, resulting in all p-values being less than .001.
FISH, fluorescence *in situ* hybridization; O, oligodendroglioma; A, astrocytoma; R^2, coefficient of determination; AUC, area under the curve.

Supplementary Table 3. List of 523 Genes for Next-generation Sequencing

Gene list	ABL1, ABL2, ACVR1, ACVR1B, AKT1, AKT2, AKT3, ALK, ALOX12B, ANKRD11, ANKRD26, APC, AR, ARAF, ARFRP1, ARID1A, ARID1B, ARID2, ARID5B, ASXL1, ASXL2, ATM, ATR, ATRX, AURKA, AURKB, AXIN1, AXIN2, AXL, B2M, BAP1, BARD1, BBC3, BCL10, BCL2, BCL2L1, BCL2L11, BCL2L2, BCL6, BCOR, BCORL1, BCR, BIRC3, BLM, BMPR1A, BRAF, BRCA1, BRCA2, BRD4, BRIP1, BTG1, BTK, C11orf30, CALR, CARD11, CASP8, CBFB, CBL, CCND1, CCND2, CCND3, CCNE1, CD274, CD276, CD74, CD79A, CD79B, CDC73, CDH1, CDK12, CDK4, CDK6, CDK8, CDKN1A, CDKN1B, CDKN2A, CDKN2B, CDKN2C, CEBPA, CENPA, CHD2, CHD4, CHEK1, CHEK2, CIC, CREBBP, CRKL, CRLF2, CSF1R, CSF3R, CSNK1A1, CTCF, CTLA4, CTNNA1, CTNNB1, CUL3, CUX1, CXCR4, CYLD, DAXX, DCUN1D1, DDR2, DDX41, DHX15, DICER1, DIS3, DNAJB1, DNMT1, DNMT3A, DNMT3B, DOT1L, E2F3, EED, EGFL7, EGFR, EIF1AX, EIF4A2, EIF4E, EML4, EP300, EPCAM, EPHA3, EPHA5, EPHA7, EPHB1, ERBB2, ERBB3, ERBB4, ERCC1, ERCC2, ERCC3, ERCC4, ERCC5, ERG, ERRFI1, ESR1, ETS1, ETV1, ETV4, ETV5, ETV6, EWSR1, EZH2, FAM123B, FAM175A, FAM46C, FANCA, FANCC, FANCD2, FANCE, FANCF, FANCG, FANCI, FANCL, FAS, FAT1, FBXW7, FGF1, FGF10, FGF14, FGF19, FGF2, FGF23, FGF3, FGF4, FGF5, FGF6, FGF7, FGF8, FGF9, FGFR1, FGFR2, FGFR3, FGFR4, FH, FLCN, FLI1, FLT1, FLT3, FLT4, FOXA1, FOXL2, FOXO1, FOXP1, FRS2, FUBP1, FYN, GABRA6, GATA1, GATA2, GATA3, GATA4, GATA6, GEN1, GID4, GLI1, GNA11, GNA13, GNAQ, GNAS, GPR124, GPS2, GREM1, GRIN2A, GRM3, GSK3B, H3F3A, H3F3B, H3F3C, HGF, HIST1H1C, HIST1H2BD, HIST1H3A, HIST1H3B, HIST1H3C, HIST1H3D, HIST1H3E, HIST1H3F, HIST1H3G, HIST1H3H, HIST1H3I, HIST1H3J, HIST2H3A, HIST2H3C, HIST2H3D, HIST3H3, HLA-A, HLA-B, HLA-C, HNF1A, HNRNPK, HOXB13, HRAS, HSD3B1, HSP90AA1, ICOSLG, ID3, IDH1, IDH2, IFNGR1, IGF1, IGF1R, IGF2, IKBKE, IKZF1, IL10, IL7R, INHA, INHBA, INPP4A, INPP4B, INSR, IRF2, IRF4, IRS1, IRS2, JAK1, JAK2, JAK3, JUN, KAT6A, KDM5A, KDM5C, KDM6A, KDR, KEAP1, KEL, KIF5B, KIT, KLF4, KLHL6, KMT2B, KMT2C, KMT2D, KRAS, LAMP1, LATS1, LATS2, LMO1, LRP1B, LYN, LZTR1, MAGI2, MALT1, MAP2K1, MAP2K2, MAP2K4, MAP3K1, MAP3K13, MAP3K14, MAP3K4, MAPK1, MAPK3, MAX, MCL1, MDC1, MDM2, MDM4, MED12, MEF2B, MEN1, MET, MGA, MITF, MLH1, MLL, MLLT3, MPL, MRE11A, MSH2, MSH3, MSH6, MST1, MST1R, MTOR, MUTYH, MYB, MYC, MYCL1, MYCN, MYD88, MYOD1, NAB2, NBN, NCOA3, NCOR1, NEGR1, NF1, NF2, NFE2L2, NFKBIA, NKX2-1, NKX3-1, NOTCH1, NOTCH2, NOTCH3, NOTCH4, NPM1, NRAS, NRG1, NSD1, NTRK1, NTRK2, NTRK3, NUP93, NUTM1, PAK1, PAK3, PAK7, PALB2, PARK2, PARP1, PAX3, PAX5, PAX7, PAX8, PBRM1, PDCD1, PDCD1LG2, PDGFRA, PDGFRB, PDK1, PDPK1, PGR, PHF6, PHOX2B, PIK3C2B, PIK3C2G, PIK3C3, PIK3CA, PIK3CB, PIK3CD, PIK3CG, PIK3R1, PIK3R2, PIK3R3, PIM1, PLCG2, PLK2, PMAIP1, PMS1, PMS2, PNRC1, POLD1, POLE, PPARG, PPM1D, PPP2R1A, PPP2R2A, PPP6C, PRDM1, PREX2, PRKAR1A, PRKCI, PRKDC, PRSS8, PTCH1, PTEN, PTPN11, PTPRD, PTPRS, PTPRT, QKI, RAB35, RAC1, RAD21, RAD50, RAD51, RAD51B, RAD51C, RAD51D, RAD52, RAD54L, RAF1, RANBP2, RARA, RASA1, RB1, RBM10, RECQL4, REL, RET, RFWD2, RHEB, RHOA, RICTOR, RIT1, RNF43, ROS1, RPS6KA4, RPS6KB1, RPS6KB2, RPTOR, RUNX1, RUNX1T1, RYBP, SDHA, SDHAF2, SDHB, SDHC, SDHD, SETBP1, SETD2, SF3B1, SH2B3, SH2D1A, SHQ1, SLIT2, SLX4, SMAD2, SMAD3, SMAD4, SMARCA4, SMARCB1, SMARCD1, SMC1A, SMC3, SMO, SNCAIP, SOCS1, SOX10, SOX17, SOX2, SOX9, SPEN, SPOP, SPTA1, SRC, SRSF2, STAG1, STAG2, STAT3, STAT4, STAT5A, STAT5B, STK11, STK40, SUFU, SUZ12, SYK, TAF1, TBX3, TCEB1, TCF3, TCF7L2, TERC, TERT, TET1, TET2, TFE3, TFRC, TGFBR1, TGFBR2, TMEM127, TMPRSS2, TNFAIP3, TNFRSF14, TOP1, TOP2A, TP53, TP63, TRAF2, TRAF7, TSC1, TSC2, TSHR, U2AF1, VEGFA, VHL, VTCN1, WISP3, WT1, XIAP, XPO1, XRCC2, YAP1, YES1, ZBTB2, ZBTB7A, ZFHX3, ZNF217, ZNF703, ZRSR2
Platform	Illumina NextSeq550Dx; TruSight™ Oncology 500

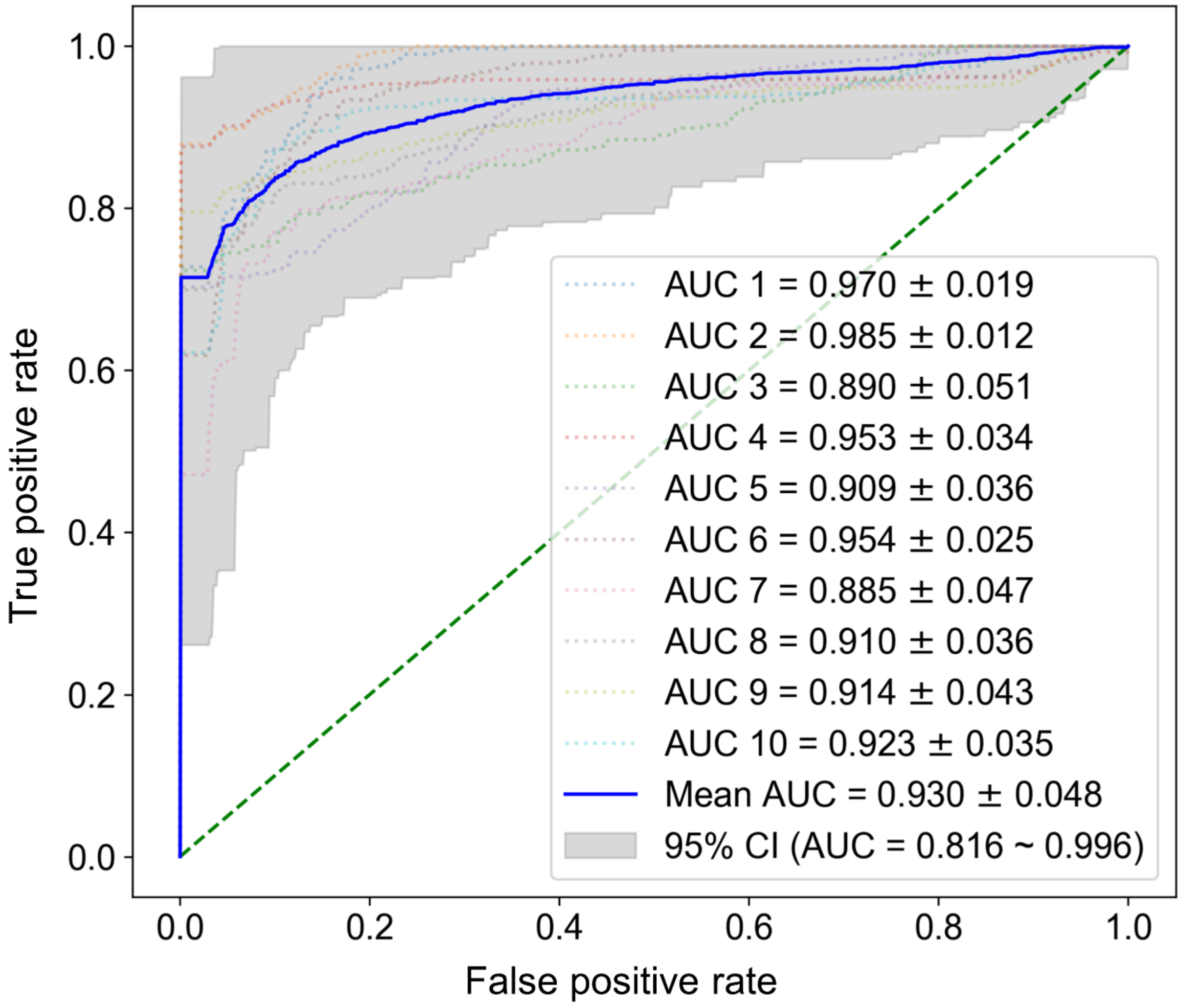

Supplementary Figure 1. Receiver Operating Characteristic Curves of 1p/19qNET on Each Fold of the Discovery Set with Bootstrap-confirmed Confidence Intervals. The findings were consistent with those depicted in Figure 2B, thereby enhancing the statistical robustness of the results. To further strengthen the reliability of our results, we employed bootstrap analysis, which allowed us to estimate the confidence interval. AUC, area under the curve; CI, confidence interval.

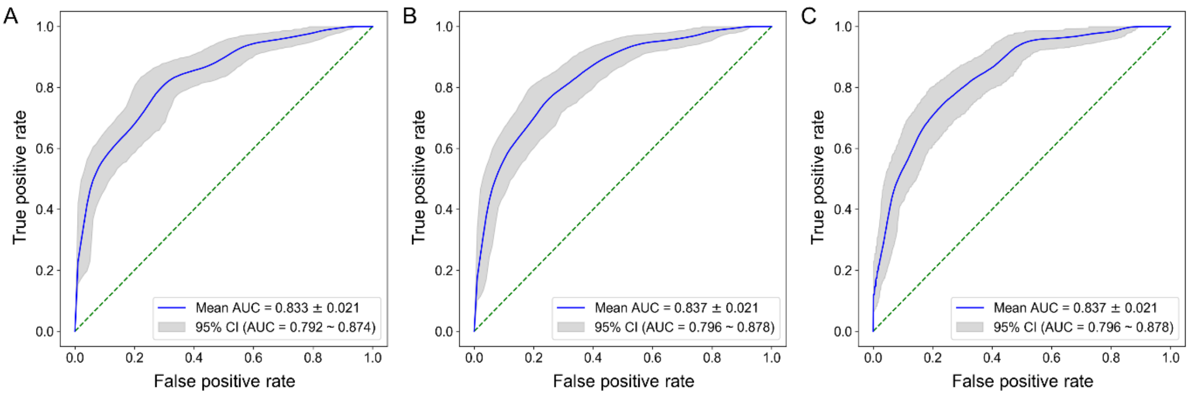

Supplementary Figure 2. Receiver Operating Characteristic Curves of 1pNET and 19qNET on the Independent Validation Set. A 1pNET. **B** 19qNET. **C** Logistic model. Each model demonstrated a good diagnostic performance, as validated by the rigorous technique of bootstrap resampling. AUC, area under the curve; CI, confidence interval.

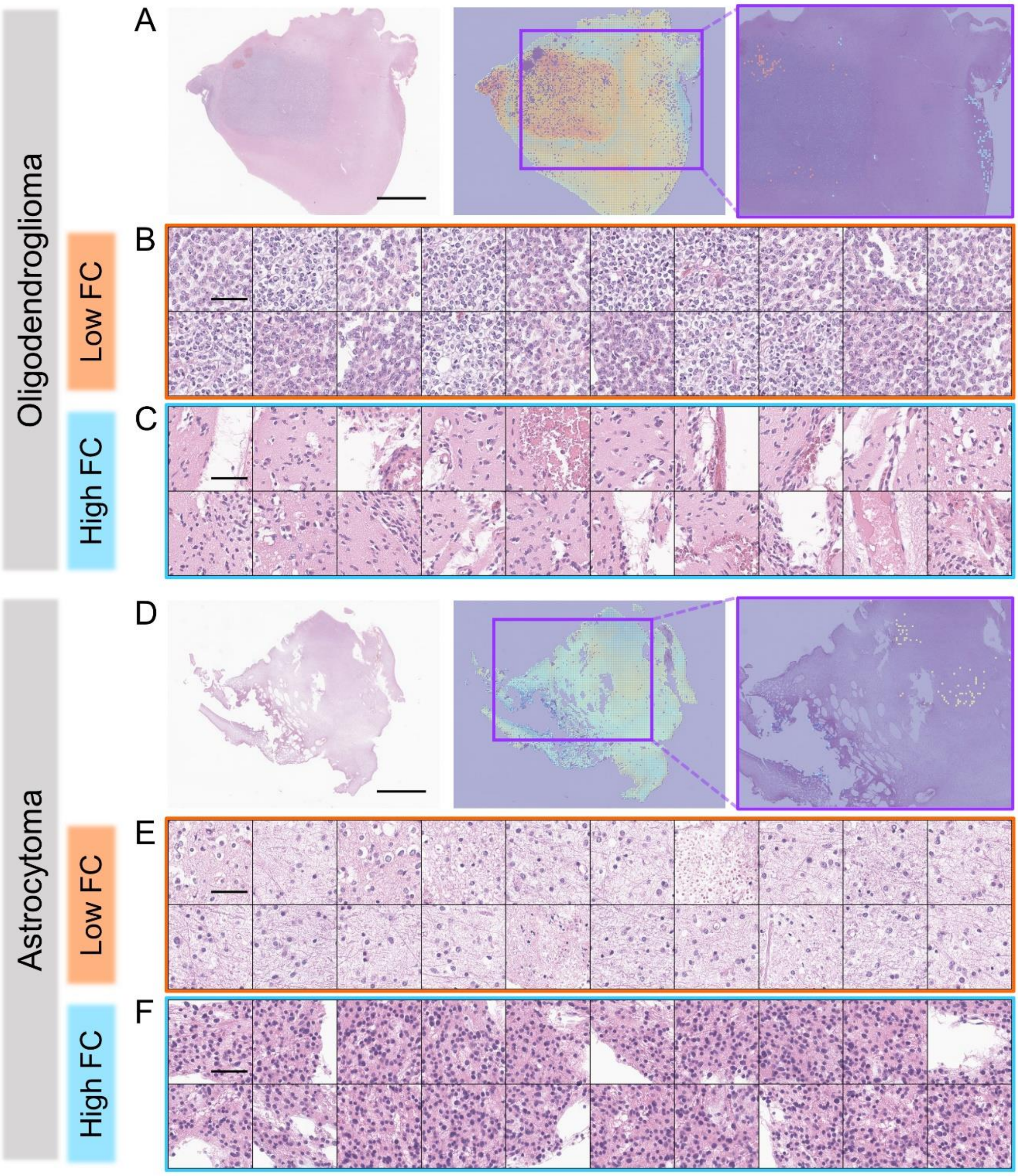

Supplementary Figure 3. Heatmaps and Representative Patches of 19qNET. A-C oligodendroglioma. **D-F** astrocytoma. **A and D** heatmap and distribution of representative patches. Scale bar, 5 mm. **B and E** 20 patches out of 100 representative patches with low FC value. Scale bar, 50 µm. **C and F** 20 patches out of 100 representative patches with high FC value. Scale bar, 50 µm. FC, fold change.

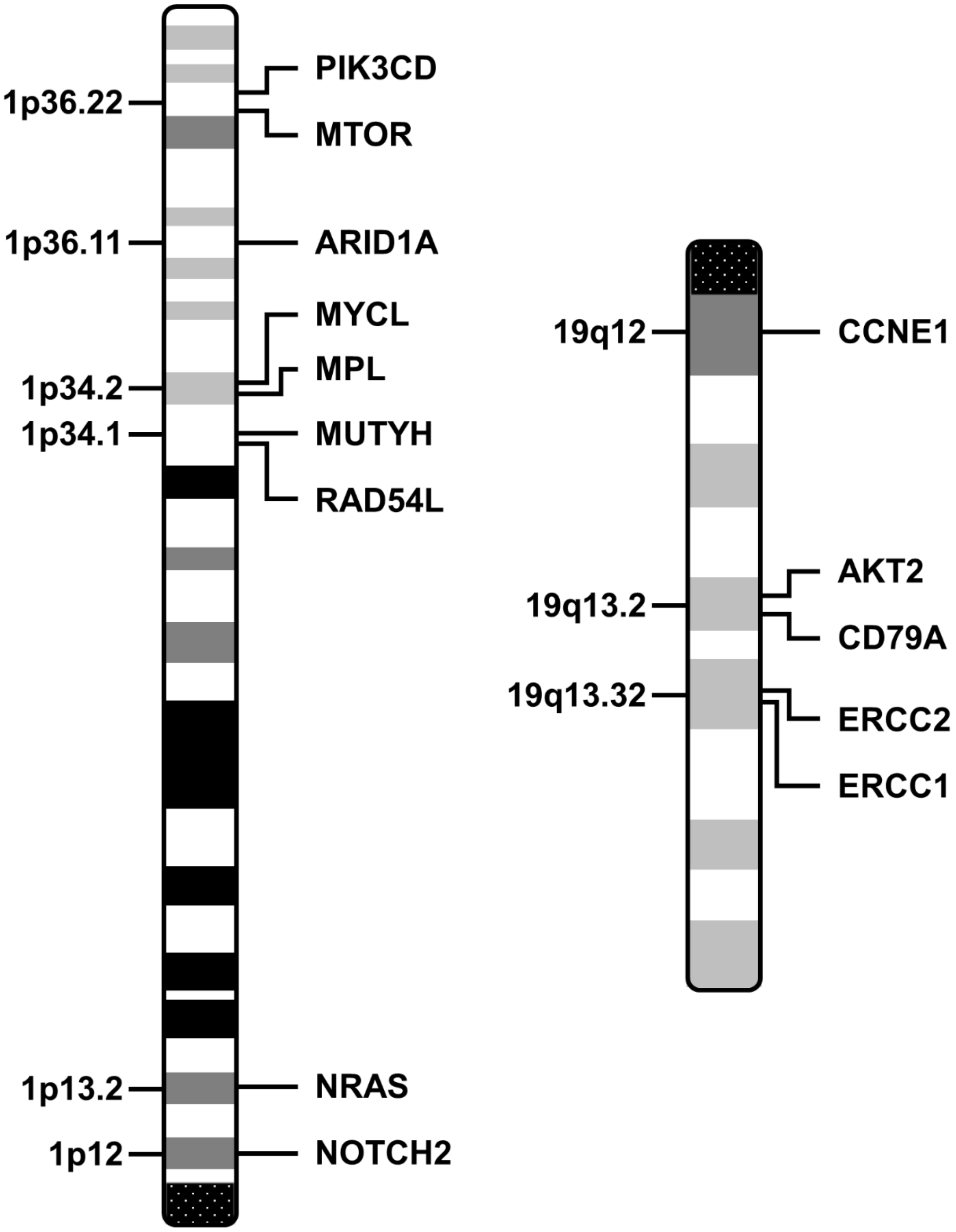

Supplementary Figure 4. Chromosomal Location of the Target Genes. The target genes were evenly distributed across the p-arm of chromosome 1 and the q-arm of chromosome 19, rendering a consistent decrease in fold change values across all the target genes on these chromosomes highly indicative of 1p/19q codeletion.

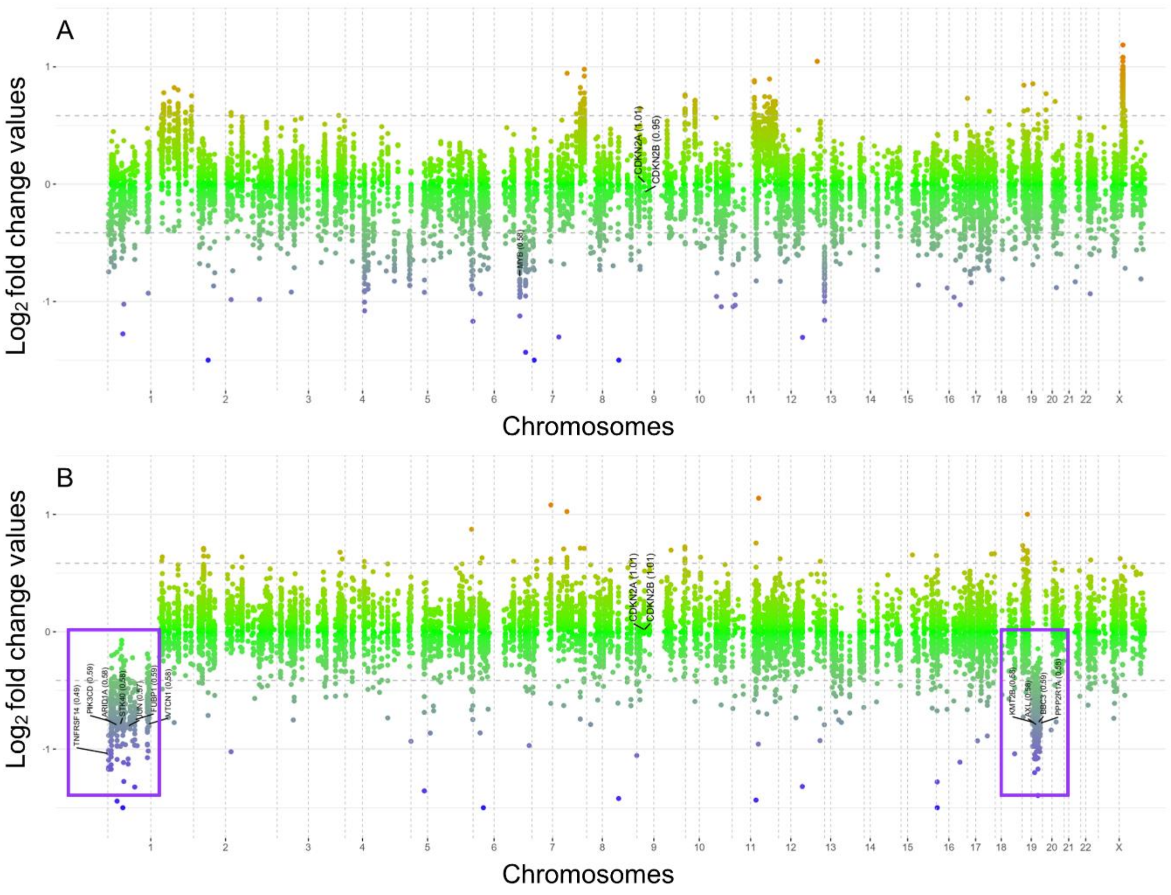

Supplementary Figure 5. Representative Copy Number Plots. A The presented astrocytoma did not show significant fold change alterations at the locations of 1p and 19q, while **B** oligodendroglioma showed a simultaneous decrease in fold change values at those locations (purple boxes). The plots were provided along with the corresponding fold change values, which improved the reliability of the diagnosis.
