# Supplementary material for: Efficient diagnosis of IDH-mutant gliomas: 1p/19qNET assesses 1p/19q codeletion status using weakly-supervised learning
Source: NPJ Precis Oncol. 2023 Sep 16;7:94. doi: 10.1038/s41698-023-00450-4 (PMC10505231; doi:10.1038/s41698-023-00450-4)
Supplement: Supplementary file 2 — Reporting Summary [file 41698_2023_450_MOESM2_ESM.pdf]

nature portfolio

Corresponding author(s): Se Hoon Kim

Last updated by author(s): Sep 1, 2023

Reporting Summary

Nature Portfolio wishes to improve the reproducibility of the work that we publish. This form provides structure for consistency and transparency in reporting. For further information on Nature Portfolio policies, see our Editorial Policies and the Editorial Policy Checklist.

Statistics

For all statistical analyses, confirm that the following items are present in the figure legend, table legend, main text, or Methods section.

n/a	Confirmed	
☐	☒	The exact sample size (*n*) for each experimental group/condition, given as a discrete number and unit of measurement
☐	☒	A statement on whether measurements were taken from distinct samples or whether the same sample was measured repeatedly
☐	☒	The statistical test(s) used AND whether they are one- or two-sided<br>*Only common tests should be described solely by name; describe more complex techniques in the Methods section.*
☒	☐	A description of all covariates tested
☒	☐	A description of any assumptions or corrections, such as tests of normality and adjustment for multiple comparisons
☐	☒	A full description of the statistical parameters including central tendency (e.g. means) or other basic estimates (e.g. regression coefficient) AND variation (e.g. standard deviation) or associated estimates of uncertainty (e.g. confidence intervals)
☐	☒	For null hypothesis testing, the test statistic (e.g. *F*, *t*, *r*) with confidence intervals, effect sizes, degrees of freedom and *P* value noted<br>*Give P values as exact values whenever suitable.*
☒	☐	For Bayesian analysis, information on the choice of priors and Markov chain Monte Carlo settings
☒	☐	For hierarchical and complex designs, identification of the appropriate level for tests and full reporting of outcomes
☐	☒	Estimates of effect sizes (e.g. Cohen's *d*, Pearson's *r*), indicating how they were calculated

Our web collection on statistics for biologists contains articles on many of the points above.

Software and code

Policy information about availability of computer code

Data collection	GDC Data Transfer Tool v 1.0.0
Data analysis	You can access the data preprocessing code in the GitHub repository located at https://github.com/Xiyue-Wang/RetCCL. Additionally, for the primary codes required to reproduce the project and further investigate the findings, please refer to https://github.com/rogo96/1p19qNET. These repositories provide the necessary code for replicating the research outcomes and conducting further explorations.

For manuscripts utilizing custom algorithms or software that are central to the research but not yet described in published literature, software must be made available to editors and reviewers. We strongly encourage code deposition in a community repository (e.g. GitHub). See the Nature Portfolio guidelines for submitting code & software for further information.

Data

Policy information about availability of data

All manuscripts must include a data availability statement. This statement should provide the following information, where applicable:

- Accession codes, unique identifiers, or web links for publicly available datasets
- A description of any restrictions on data availability
- For clinical datasets or third party data, please ensure that the statement adheres to our policy

We conducted independent validation using a publicly available dataset accessible through the TCGA portal (https://portal.gdc.cancer.gov). Specifically, we utilized data from the TCGA-LGG and TCGA-GBM projects. The primary dataset that underpins the findings of this study is sourced from Severance Hospital. However, it's

important to note that access to this dataset is subject to restrictions, and its use in this study was permitted with specific authorization. As a result, this dataset is not accessible to the public. If required, access to the data or a subset for testing purposes may be granted by Severance Hospital pending the approval of ethical considerations.

Research involving human participants, their data, or biological material

Policy information about studies with human participants or human data. See also policy information about sex, gender (identity/presentation), and sexual orientation and race, ethnicity and racism.

Reporting on sex and gender	Not appplicable
Reporting on race, ethnicity, or other socially relevant groupings	Not appplicable
Population characteristics	Not appplicable
Recruitment	Not appplicable
Ethics oversight	This study was approved by the Institutional Review Board of Severance Hospital, Seoul, Korea, with the waiver for written informed consent (IRB no. 4-2022-1493).

Note that full information on the approval of the study protocol must also be provided in the manuscript.

Field-specific reporting

Please select the one below that is the best fit for your research. If you are not sure, read the appropriate sections before making your selection.

☒ Life sciences ☐ Behavioural & social sciences ☐ Ecological, evolutionary & environmental sciences

For a reference copy of the document with all sections, see nature.com/documents/nr-reporting-summary-flat.pdf

Life sciences study design

All studies must disclose on these points even when the disclosure is negative.

Sample size	No sample-size calculation was performed
Data exclusions	Cases in the TCGA-LGGGBM dataset that did not have a diagnostic permanent slide, but only frozen slides, were excluded from the analysis.
Replication	Not appplicable
Randomization	The data underwent randomization using a Python coding.
Blinding	Following the assignment of research numbers, the experiments were conducted and the results were analyzed.

Reporting for specific materials, systems and methods

We require information from authors about some types of materials, experimental systems and methods used in many studies. Here, indicate whether each material, system or method listed is relevant to your study. If you are not sure if a list item applies to your research, read the appropriate section before selecting a response.

Materials & experimental systems

n/a	Involved in the study	
☒	☐	Antibodies
☒	☐	Eukaryotic cell lines
☒	☐	Palaeontology and archaeology
☒	☐	Animals and other organisms
☒	☐	Clinical data
☒	☐	Dual use research of concern
☒	☐	Plants

Methods

n/a	Involved in the study	
☒	☐	ChIP-seq
☒	☐	Flow cytometry
☒	☐	MRI-based neuroimaging
